# Unintended Pregnancy in Ethiopia: Community Based Cross-Sectional Study

**DOI:** 10.1155/2016/4374791

**Published:** 2016-08-30

**Authors:** Kidest Getu Melese, Mignote Hailu Gebrie, Martha Berta Badi, Wubalem Fekadu Mersha

**Affiliations:** ^1^Department of Midwifery, College of Health Sciences and Medicine, Wolaita Sodo University, Wolaita Sodo, Ethiopia; ^2^Department of Nursing, College of Medicine and Health Sciences, University of Gondar, Gondar, Ethiopia; ^3^Department of Midwifery, College of Medicine and Health Sciences, University of Gondar, Gondar, Ethiopia; ^4^Department of Psychiatry, College of Health Sciences and Medicine, Bahir Dar University, Bahir Dar, Ethiopia

## Abstract

*Introduction*. Unintended pregnancy is defined as a pregnancy which is a sum of mistimed pregnancy (pregnancy wanted at a later time) and unwanted pregnancy (pregnancy which is not wanted at all). Unintended pregnancy is a global public health problem and its sequels are major causes for maternal and neonatal morbidity and mortality with its effect to maternal metal illness as well.* Objective*. To determine the prevalence and associated factors of unintended pregnancy in Debre Birhan town, northeast of Ethiopia, in 2014.* Method*. Community based cross-sectional study and questionnaire developed from Ethiopian demographic health survey 2011. Participants were 690 currently pregnant mothers. Association of unintended pregnancy with factors was measured with bivariate and multivariate logistic regressions.* Result*. In this study unintended pregnancy is found to be 23.5%. Being formerly married and never married, distance to the nearest health facility >80 minutes, gravidity >5, 1-2 parity, and partner disagreement on desired number of children are the variables significantly associated with unintended pregnancy.* Conclusion*. Significant proportion of unintended pregnancy is found in the study area. To minimize unintended pregnancy concerned bodies should work on the identified factors, so we can minimize maternal and neonatal morbidity and mortality and keep the health of the family specifically and country in general.

## 1. Background

Unintended pregnancy is defined as pregnancy, which admits either mistimed or unwanted pregnancy [1, 2]. It is a major global public health problem with bad complication for a mother, a child, a family, a society, and a country as a whole [1, 3–6]. Unintended pregnancy has direct relation with poor utilization of maternal health care services. Researches relate unintended pregnancy with poor antenatal care utilization, which is a risk factor for unfavorable pregnancy outcome and maternal morbidity and mortality [7–9]. In addition unintended pregnancy results in unsafe abortion, which is the other main cause of maternal mortality and morbidity. Nearly 80,000 mothers die per year because of unsafe abortion and almost 95% of deaths happen in developing countries [10].

Unintended pregnant women will have low physical and mental health, low self-care, poor health, high level of substance addiction, and depression during pregnancy [3, 11, 12]. Besides, fetus will be delivered by unskilled attendant, delivered as low birth weight, increased rate of hospitalization, and poor growth and inadequate immunization adversely leads to maternal and child death [2, 4].

In 2012 worldwide 80 million mothers had unintended pregnancy [13]. Regionally, in more developed regions it is revealed to be 42 per 1000 women. In less developed regions from 1000 pregnancies 57 of them were unintended and the pregnancies end up with unplanned birth, abortion, and miscarriage.

Unintended pregnancy is 36 percent higher in developing country than developed country. In Africa from a total of 49.1 million pregnancies, 39% were unintended pregnancy [14]. In Ethiopia according to Ethiopian Demographic Health Survey (EDHS) 2011, the percentage of unwanted births decreased (17% in 2000 to 9% in 2011). However the percentage of pregnancy wanted later does not show improvement (19-20%) [15].

Major factors influencing unintended pregnancy are young or old age, low maternal education, unmarried marital status, low income, high parity and gravidity, long estimated time to walk to the nearest health facility, and ever use of family planning (FP) [5, 16–20].

Unintended pregnancy problems and complications are vast. However, evidences and literatures on the problem are limited. Therefore, this paper determines the proportion and associated factors of unintended pregnancy among currently pregnant women in Ethiopia.

## 2. Methods

Community based cross-sectional study was carried out in northeast, located 130 km northeast of capital city of Ethiopia, Addis Ababa. Six hundred ninety-seven currently pregnant women in study area were included in the study where stratified cluster sampling technique was used to select the study participants.

Pretested and structured interviewer administered questionnaire, which is adopted from EDHS (Ethiopian Demographic Health Survey), were used to assess pregnancy intention. Participants were considered as having unintended pregnancy if they report their current pregnancy as either mistimed or unwanted at the time of conception.

There were a total of 17 questions which is first prepared in English and then translated to local language Amharic and then translated back to English for consistency that contains three parts which assesses the sociodemographic and economic status including age, residence, marital status, education, occupation, estimated time to walk to the nearest health facility, and income. Reproductive variables include gravidity, parity, ideal number of child, and partner desire for a child. Contraceptive characteristics assess ever use of family planning. To assess pregnancy intention mothers were asked a question for the current pregnancy: when you got pregnant did you want to get pregnant at the time? Pregnancy is taken as intended if the response is yes. If no, to determine mistimed and unwanted pregnancy, did you want to wait until later or did you not want later any more children?

Foremost data completeness was checked manually, coded, entered, and analyzed into SPSS version 20. Tables 1 and 2 and frequency were used to report the descriptive result, since the dependent variable is categorical binary and multiple logistic regressions were equipped to identify associated factors of unintended pregnancy. In binary logistic regression variables with *P* value ≤ 0.2 were transferred to multiple logistic regressions to adjust the effect of confounders and to distinguish the associated factors. To determine the significance, odds ratio with confidence interval 95% and *P* value ≤ 0.05 was used.

### 2.1. Ethical Consideration

Ethical clearance was obtained from the Ethical Review Committee of University of Gondar. Permission letter was obtained from local health department. For all study participants information was given about the study before the data collection on its possible risk, benefit, confidentiality, privacy, its voluntary activity, right of withdrawal, and the time the questionnaire will take and then verbal consent was obtained. Privacy and confidentiality were kept; name of the mother was not asked and recorded.

## 3. Result and Discussion

### 3.1. Sociodemographic and Economic Characteristics

A total of 697 participants were interviewed; of them 465 (67.4%) were in the age group of 25–34. Four hundred sixty-six (67.5%) resided in urban area and 523 (75.8%) were currently married. Besides, majority of them are at tertiary education level (290) (42%) and are government and private employees (257) (37.3%). Five hundred forty-seven (79.3%) study participants claim to travel <40 minutes to reach the nearest health facility (Table 1).

### 3.2. Reproductive and Contraceptive Utilization Characteristics

Three hundred eighteen (46.1%) have 3-4 gravidity and 394 (57.1%) have 1-2 parity, respectively. Besides 367 (53.2%) have 3-4 ideal numbers of children. Moreover 229 (33.2%) married participants partners agree on the number of children. In addition 447 (64.8%) study participants had ever used FP (Table 2).

### 3.3. Proportion of Unintended Pregnancy

From a total of 690 pregnant mothers 162 (23.5%) (95% CI (20.3, 26.8)) participants insured their current pregnancy as unintended; from those 73 (10.58%) are unwanted pregnancies and (the rest) 89 (12.9%) are mistimed pregnancies.

From the mentioned reasons for failure to avoid unwanted and mistimed pregnancy, contraceptive failure (24.66%) was the major reasons for unwanted pregnancy whereas for mistimed pregnancy not using FP (42%) takes the highest percentage.

### 3.4. Factors Associated with Unintended Pregnancy

In multivariable logistic regression being formerly married and never married, estimated time to walk to the nearest health facility >80 minutes, gravidity ≥5, 1-2 parity, and partner disagreement were significantly associated with unintended pregnancy (Table 3).

## 4. Discussion

Proportion of unintended pregnancy is 23.5% (95% CI (20.3, 26.8)), which is in line with the national study conducted in Ethiopia (24%) [5]. However it is slightly lower than the result reported in the national figure EDHS 2011 which is 29% [4] and also lower than the studies in different parts of Ethiopia, in Kersa Woreda, eastern Ethiopia, Harar, Hosanna, and Ganji Woreda, West Welega, Oromia which is 27%, 34%, and 36.5%, respectively [16, 17, 21] and study in Tanzania (50.7%) [7]. The result might be lower due to the fact that once pregnancy happened there is tendency to be confirmed as intended [22]. In addition the variance might be on account of the difference in sociodemographic and cultural status of the study areas.

The odds of having unintended pregnancy among formerly and never married women are 8.42 and 9.21 times more likely than currently married women. This finding is consistent with the study in Nairobi, Kenya [23]. Women who travel >80 minutes to health facility are 3.56 times more likely to report their pregnancy as unintended; this may be due to less supply of family planning service [24]. The finding is in line with study in Kersa Woreda Eastern Ethiopia [16].

Women whose gravidity ≥5 are 3.88 times more likely to have unintended pregnancy than those whose gravidity 1-2, which is supported by other studies [17]. Women having 1-2 live births were less likely to have unintended pregnancy as compared to their counterparts. This finding is in line with the study in Kersa Woreda eastern Ethiopia and Senegal, which states association of high parity (7+) and (2+) with unintended pregnancy [16, 18]. Partner desire for child shows significant association with unintended pregnancy. This is consistent with the study conducted in Hosanna town and report in Ganji Woreda, West Welega, Oromia. The possible reason mentioned in those towns and in this area is the difference in men and women interest in number of children and men want more child than women; socioculturally child is seen as wealth in the community [17, 21].

## 5. Conclusions and Recommendations

Proportion of unintended pregnancy in the study area was high. Formerly and never married respondents, distance to the nearest health facility >80 minutes, ≥5 gravidity, 1-2 parity, and partner disagreement on desired number of children were significantly associated with the outcome variable. Therefore, high effort needed to be exerted on this target group by counseling, health education, and providing contraceptive service and clinician must consider these factors in maternal care settings.

## Figures and Tables

**Table 1:** Sociodemographic and economic characteristics of participants (*n* = 690).

Characteristics	Frequency	Percentage
Age		
≤24	112	16.2
25–34	465	67.4
≥35	113	16.4

Residence		
Urban	466	67.5
Rural	224	32.5

Marital status		
Currently married	523	75.8
Formerly married^*∗*^	76	11.0
Never married	91	13.2

Education level		
Tertiary	290	42.0
Secondary	208	30.1
Primary	113	16.4
No education	79	11.4

Monthly income in United States dollar		
≥68.66	456	66.1
45.79–68.62	125	18.1
22.92–45.75	76	11.0
≤22.87	33	4.8

Occupation		
Government employee	238	34.5
Private employee	19	2.8
Farmer	133	19.3
Merchant	89	12.9
Daily labor	85	12.3
Student	32	4.6
Housewife	94	13.6

Time to walk to the nearest health facility		
<40 minutes	547	79.3
40–79 minutes	76	11.0
>80 minutes	67	9.7

Formerly married^*∗*^: divorced, widowed, and separated.

1 Ethiopian birr = 0.045746 United States dollar.

**Table 2:** Reproductive and contraceptive utilization characteristics of participants (*n* = 690).

Characteristics	Frequency	Percentage
Gravidity		
1-2	282	40.9
3-4	318	46.1
≥5	90	13.0

Parity		
0	134	19.4
1-2	394	57.1
3-4	133	19.3
≥5	29	4.2

Ideal number of children		
5–7	119	17.2
3-4	367	53.2
0–2	204	29.6

Partner desire for a child		
Agree	229	33.2
Do not know	171	24.8
Husband wants fewer	12	1.7
Husband wants more	111	16.1
Ever use of FP		
Yes	447	64.8
No	243	35.2

**Table 3:** Logistic regression analysis on factors associated with unintended pregnancy (*n* = 690).

Variables	Status of current pregnancy		COR (95% CI)	AOR (95% CI)	*P* value
	Intended	Unintended			
Age					
25–34	383 (82.4%)	82 (17.6%)	1		
≤24	69 (61.6%)	43 (38.4%)	2.91 (1.86, 4.56)	1.15 (0.56, 2.36)	0.71
≥35	76 (67.3%)	37 (32.7%)	2.27 (1.44, 3.60)	1.83 (1.00, 3.30)	0.04

Marital status					
Currently married	442 (84.5%)	81 (15.5%)	1		
Formerly married	39 (51.3%)	37 (48.7%)	5.18 (3.12, 8.61)	8.42 (4.07, 17.39)	<0.01
Never married	47 (51.6%)	44 (48.4%)	5.11 (3.18, 8.21)	9.21 (4.27, 19.86)	<0.01

Education status					
Tertiary	235 (81.0%)	55 (19.0%)	1		
Secondary	161 (77.4%)	47 (22.6%)	1.25 (0.81, 1.93)	1.2 (0.71, 2.02)	0.50
Primary	80 (70.8%)	33 (29.2%)	1.76 (1.07, 2.91)	1.21 (0.62, 2.35)	0.59
No education	52 (65.8%)	27 (34.2%)	2.22 (1.28, 3.85)	0.97 (0.42, 2.23)	0.95

Income in United States dollar					
≥68.66	348 (76.3%)	108 (23.7%)	1		
45.79–68.62	103 (82.4%)	22 (17.6%)	0.69 (0.41, 1.14)	0.75 (0.40, 1.41)	0.37
22.92–45.75	62 (81.6%)	14 (18.4%)	0.73 (0.39, 1.35)	1.00 (0.47, 2.15)	1.00
≤22.87	15 (45.5%)	18 (54.5%)	3.87 (1.89, 7.93)	1.74 (0.70, 4.33)	0.23

Time to walk to the nearest health facility					
<40 minutes	436 (79.7%)	111 (20.3%)	1		
40–79 minutes	59 (77.6%)	17 (22.4%)	1.13 (0.64, 2.02)	1.08 (0.49, 2.35)	0.86
>80 minutes	33 (49.3%)	34 (50.7%)	4.05 (2.40, 6.82)	3.56 (1.69, 7.53)	<0.01

Gravidity					
1-2	221 (78.4%)	61 (21.6%)	1		
3-4	264 (83.0%)	54 (17.0%)	0.74 (0.49, 1.11)	1.35 (0.66, 2.76)	0.41
≥5	43 (47.8%)	47 (52.2%)	3.96 (2.40, 6.54)	3.88 (1.41, 10.69)	0.01

Parity					
0	90 (67.2%)	44 (32.8%)	1		
1-2	346 (87.8%)	48 (12.2%)	0.28 (0.18, 0.45)	0.43 (0.21, 0.87)	0.02
3-4	82 (61.7%)	51 (38.3%)	1.27 (0.77, 2.10)	1.08 (0.41, 2.82)	0.88
≥5	10 (34.5%)	19 (65.5%)	3.89 (1.67, 9.06)	1.80 (0.44, 7.36)	0.41

Partner desire for child					
Agree	211 (92.1%)	18 (7.9%)	1		
Do not know	150 (87.7%)	21 (12.3%)	1.64 (0.85, 3.19)	0.66 (0.30, 1.46)	0.30
Disagree	81 (65.9%)	42 (34.1%)	6.08 (3.31, 11.17)	4.09 (2.07, 8.08)	<0.01

Ever use of FP					
Yes	363 (81.2%)	84 (18.8%)	1		
No	165 (67.9%)	78 (32.1%)	2.04 (1.43, 2.93)	1.53 (0.97, 2.41)	0.07

## Additional Points

*Limitations of the Study*. The result of the study may be underestimated because once pregnancies happen there is a tendency to be confirmed as intended.
